# Supplementary material for: Molecular analysis of archival diagnostic prostate cancer biopsies identifies genomic similarities in cases with progression post‐radiotherapy, and those with de novo metastatic disease
Source: Prostate. 2024 Apr 23;84(10):977–90. doi: 10.1002/pros.24715 (PMC11253896; doi:10.1002/pros.24715)
Supplement: Supplementary file 1 — Supporting information. [file PROS-84-977-s003.docx]

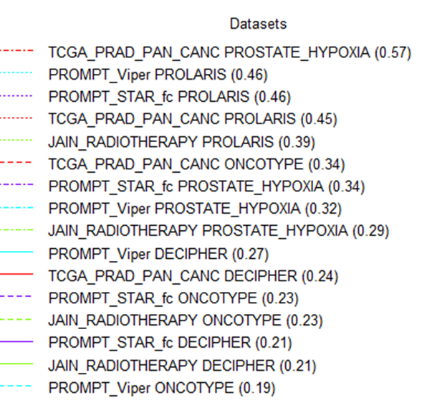

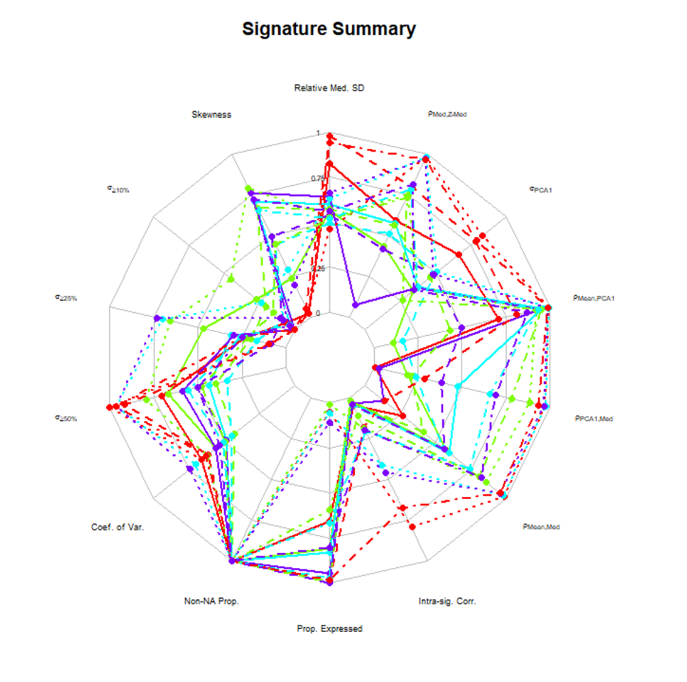


**Figure S1**


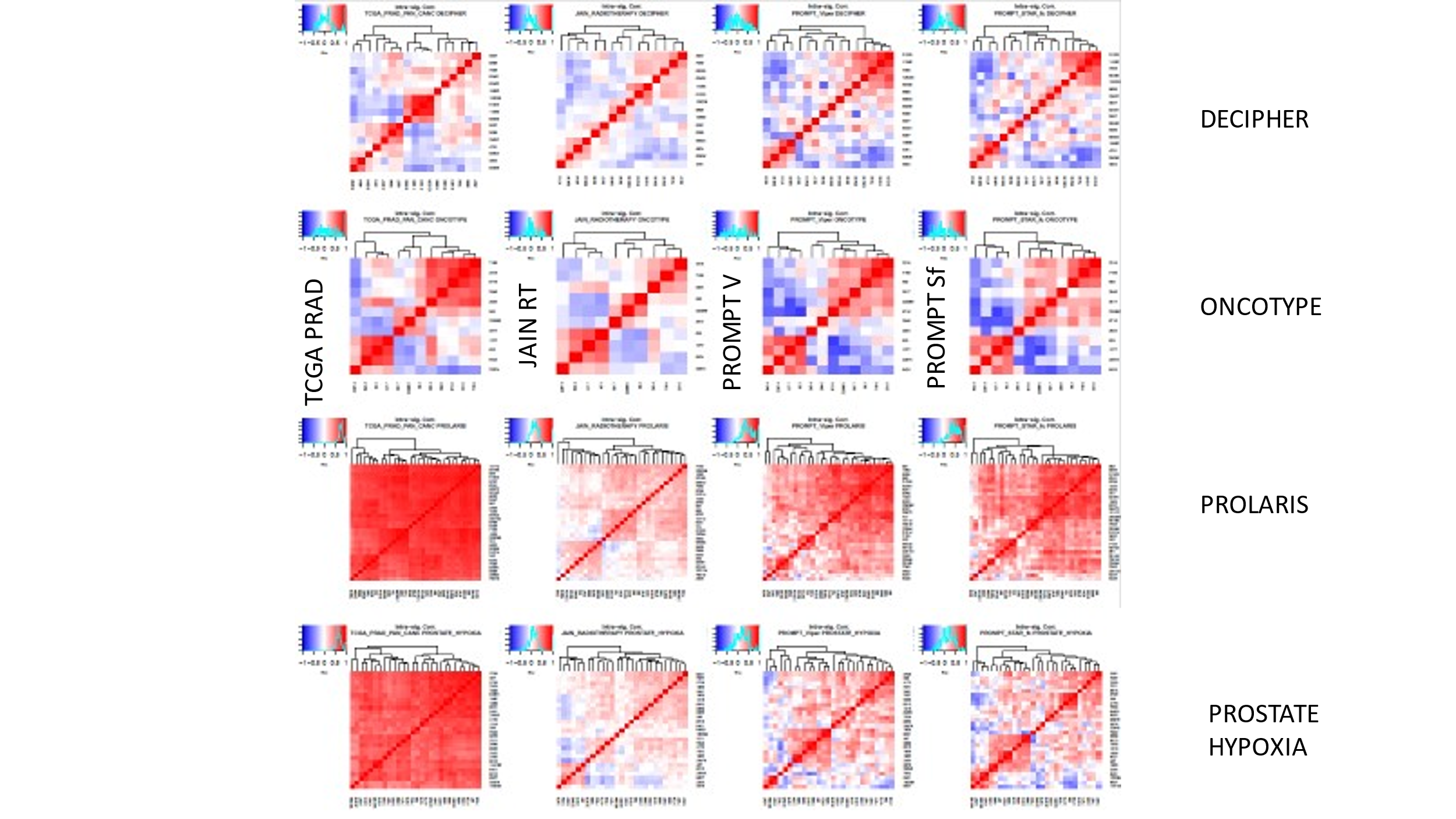


**Figure S2**


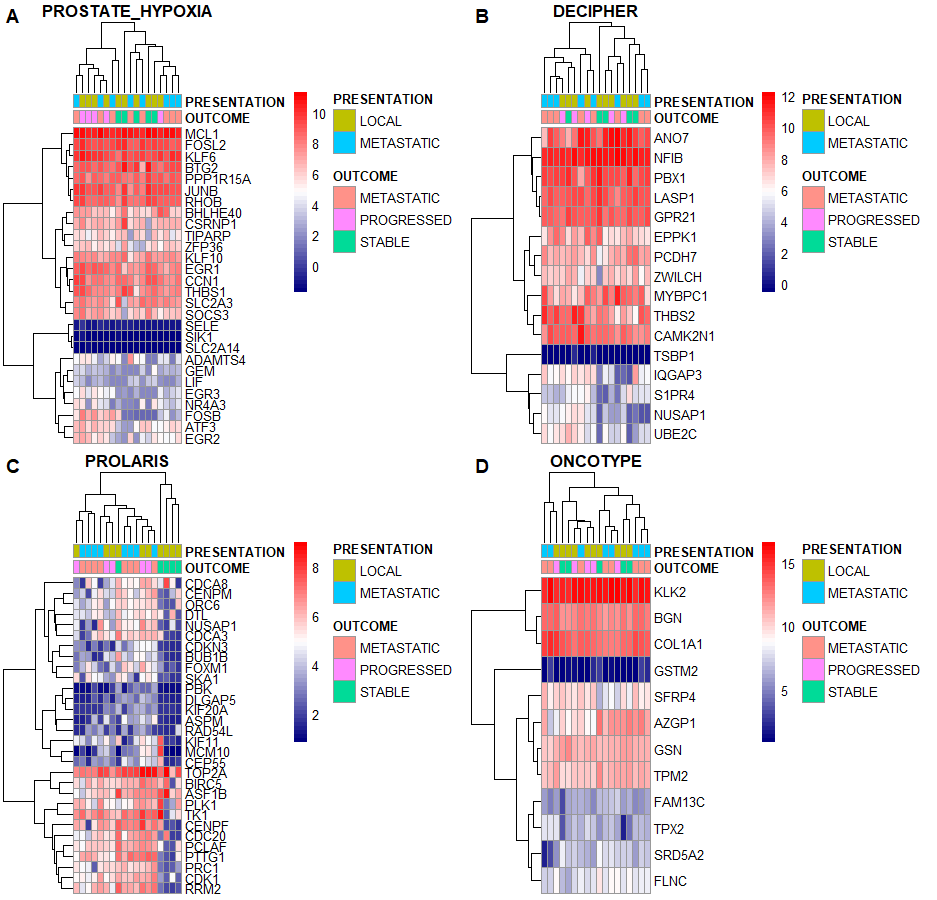


**Figure S3**

**A**

**B**

**C**

**D**


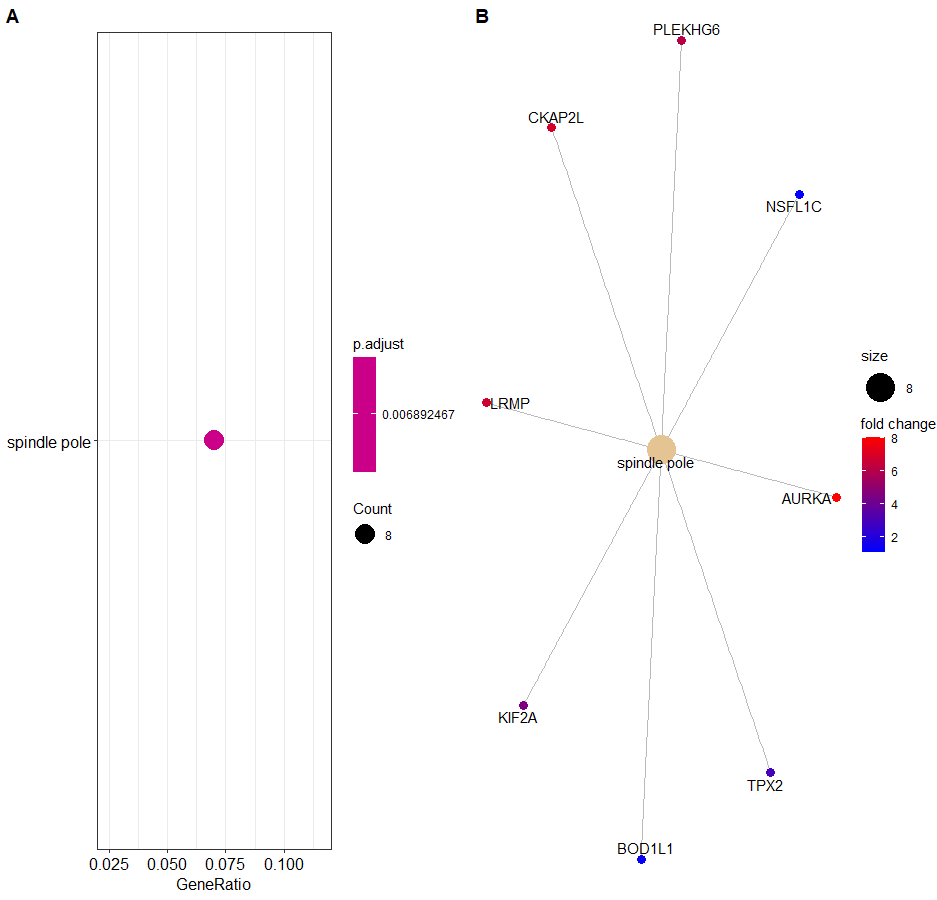


**Figure S4**

**A**

**B**


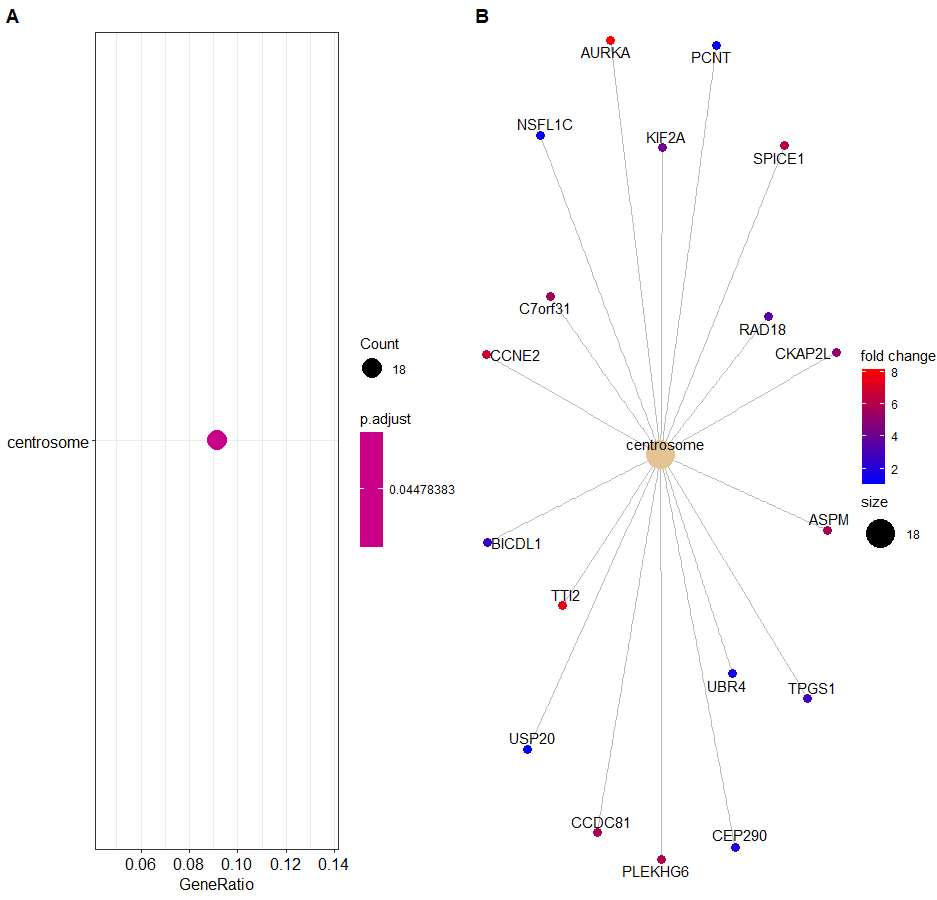


**Figure S5**

**A**

**B**

**A**

**B**


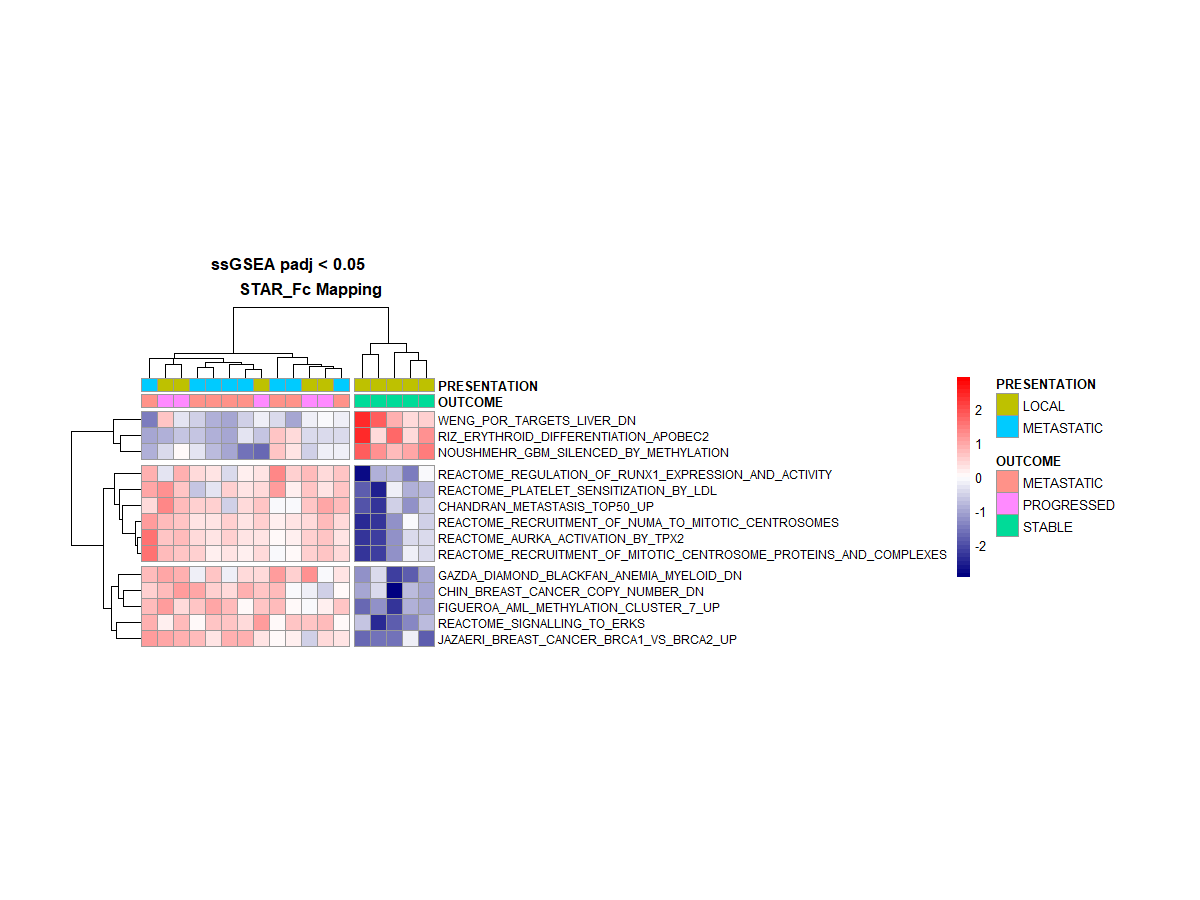


**Figure S6**


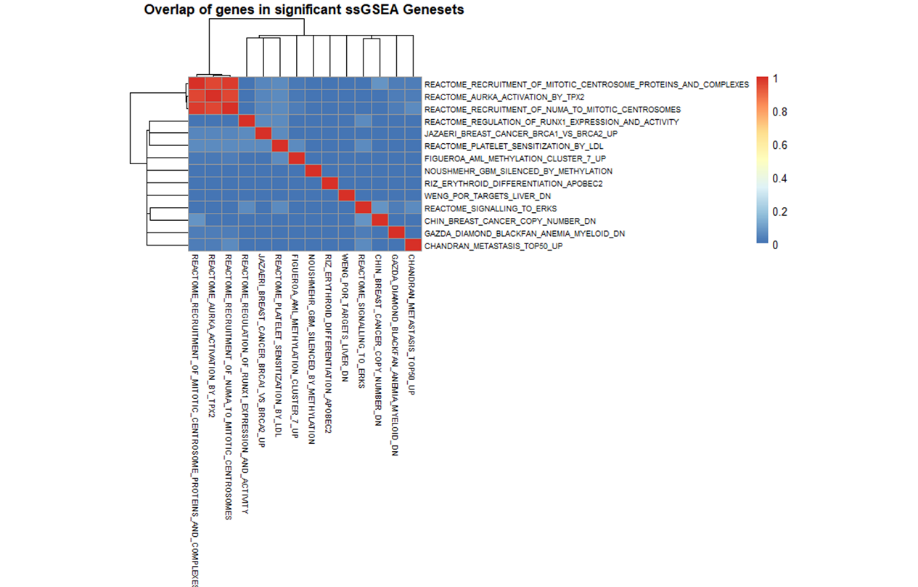


**Figure S7**


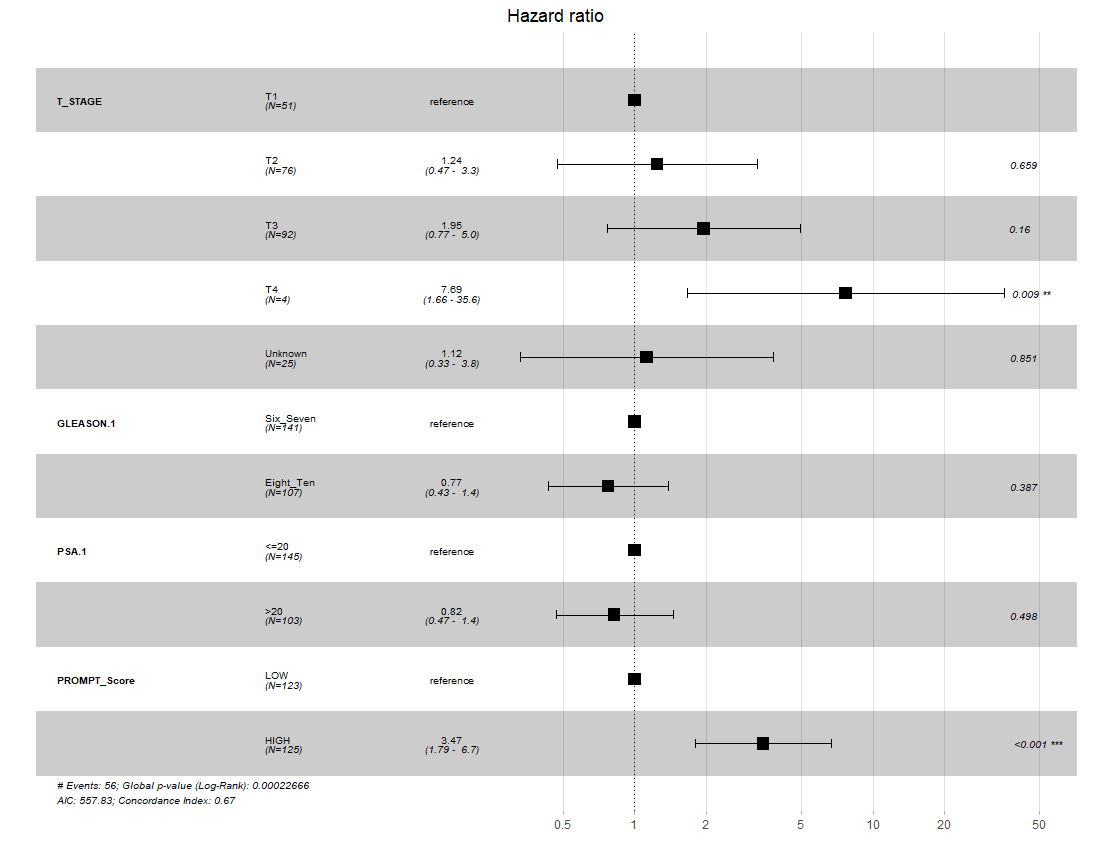


**Figure S8**

**A**

**B**

**Multi-variable Analysis**

**Uni-variable Analysis**


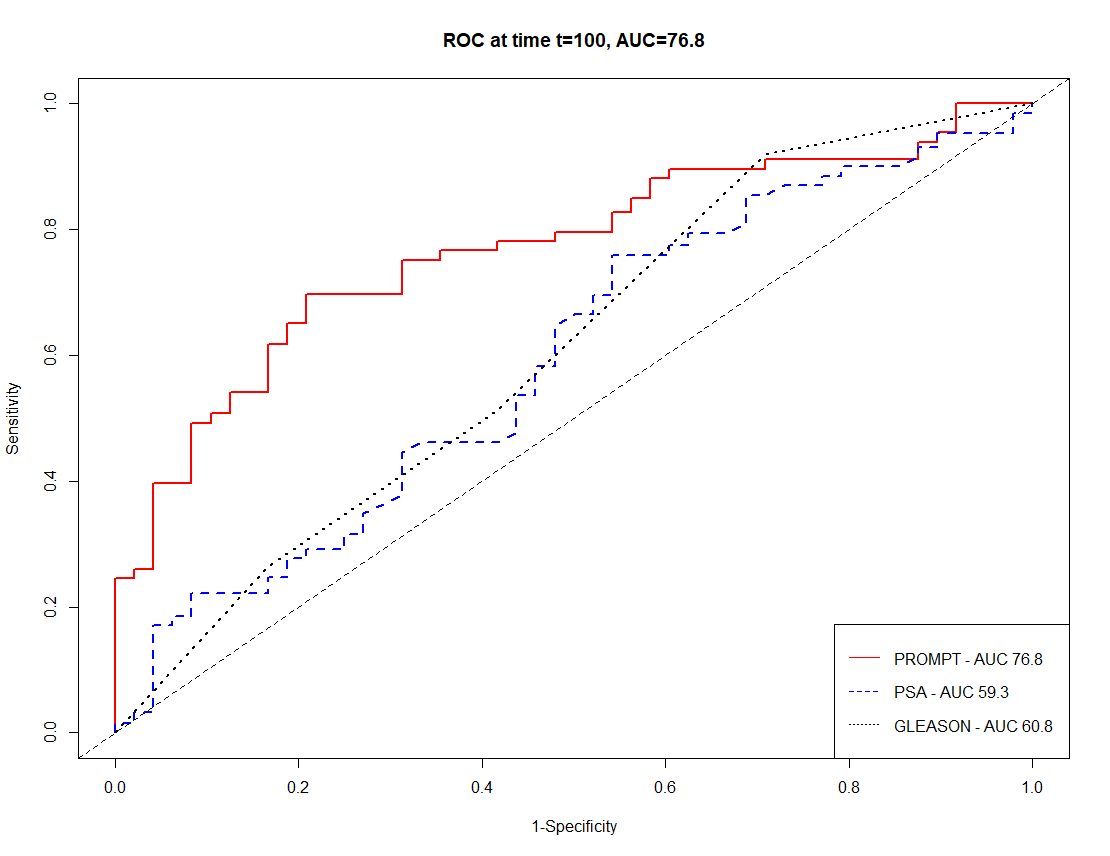

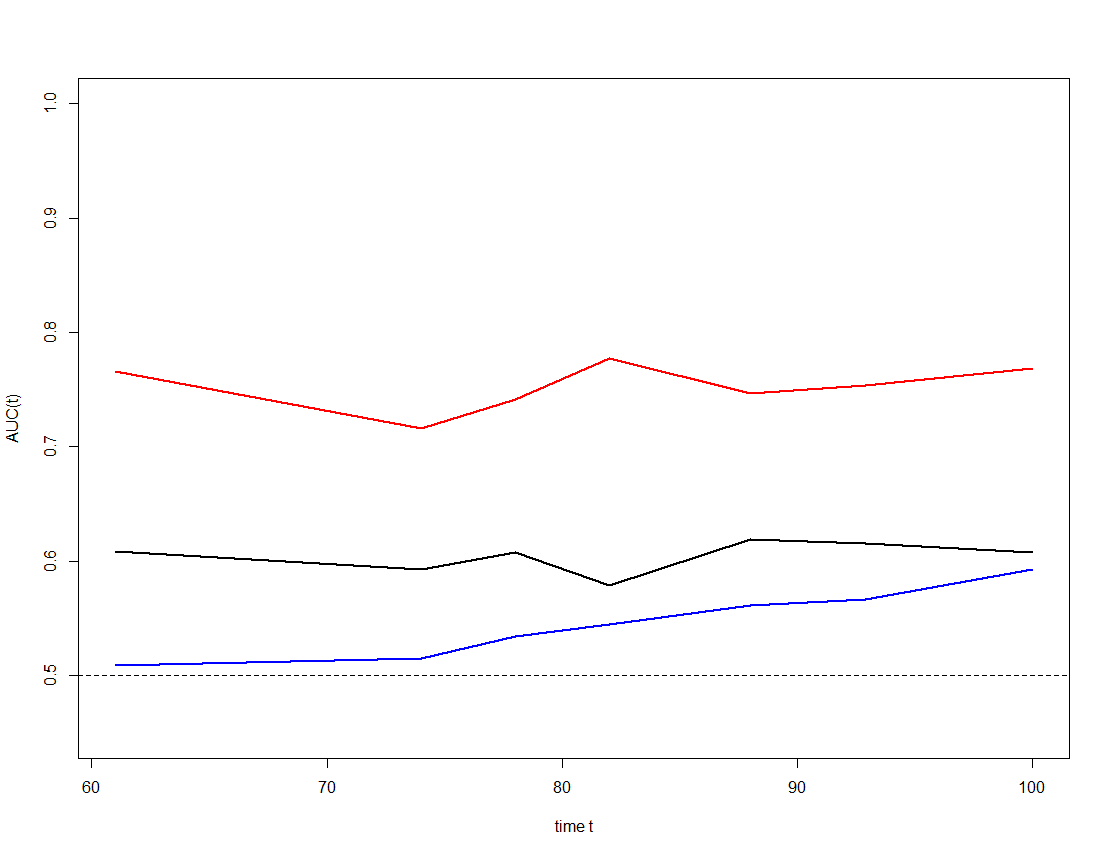

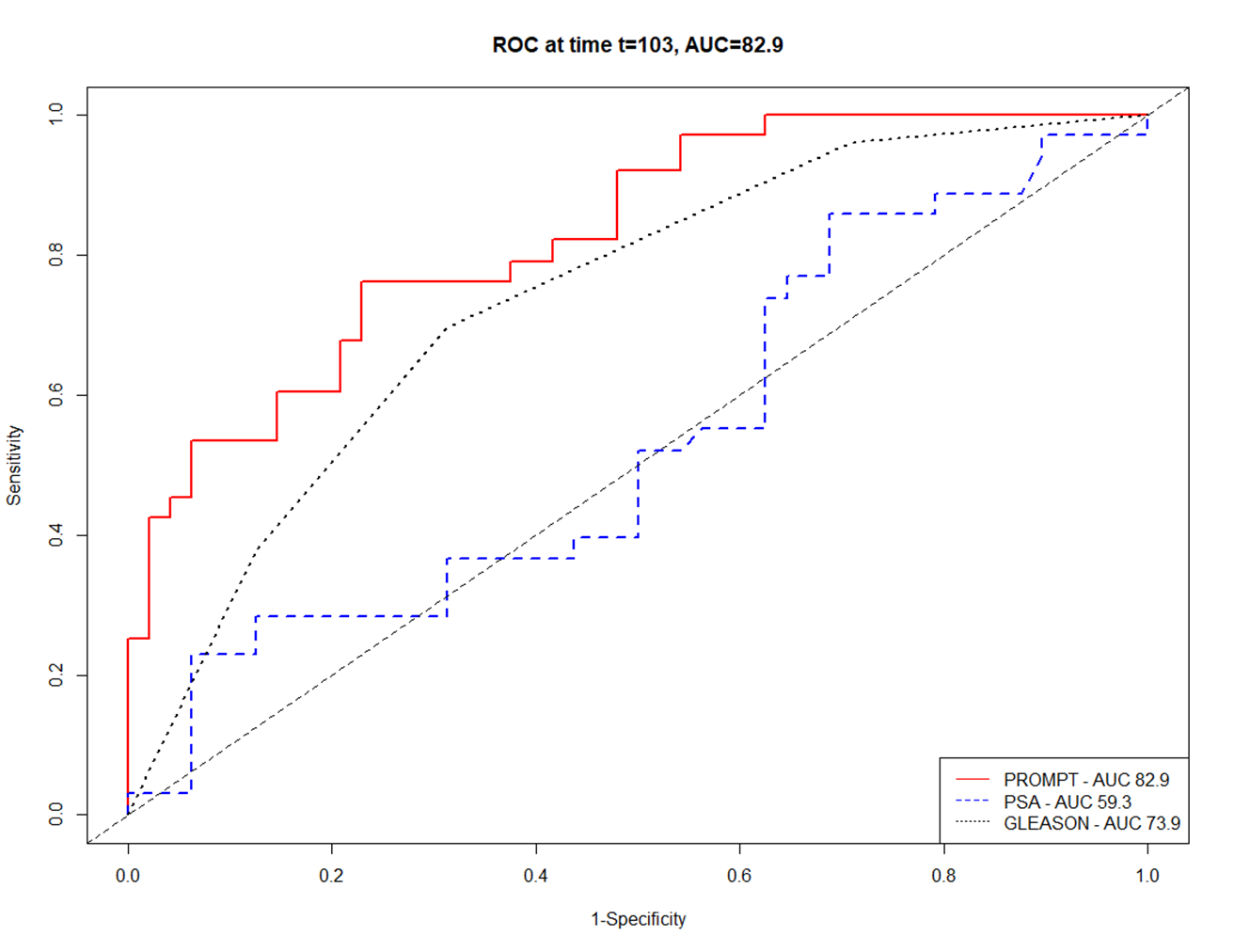

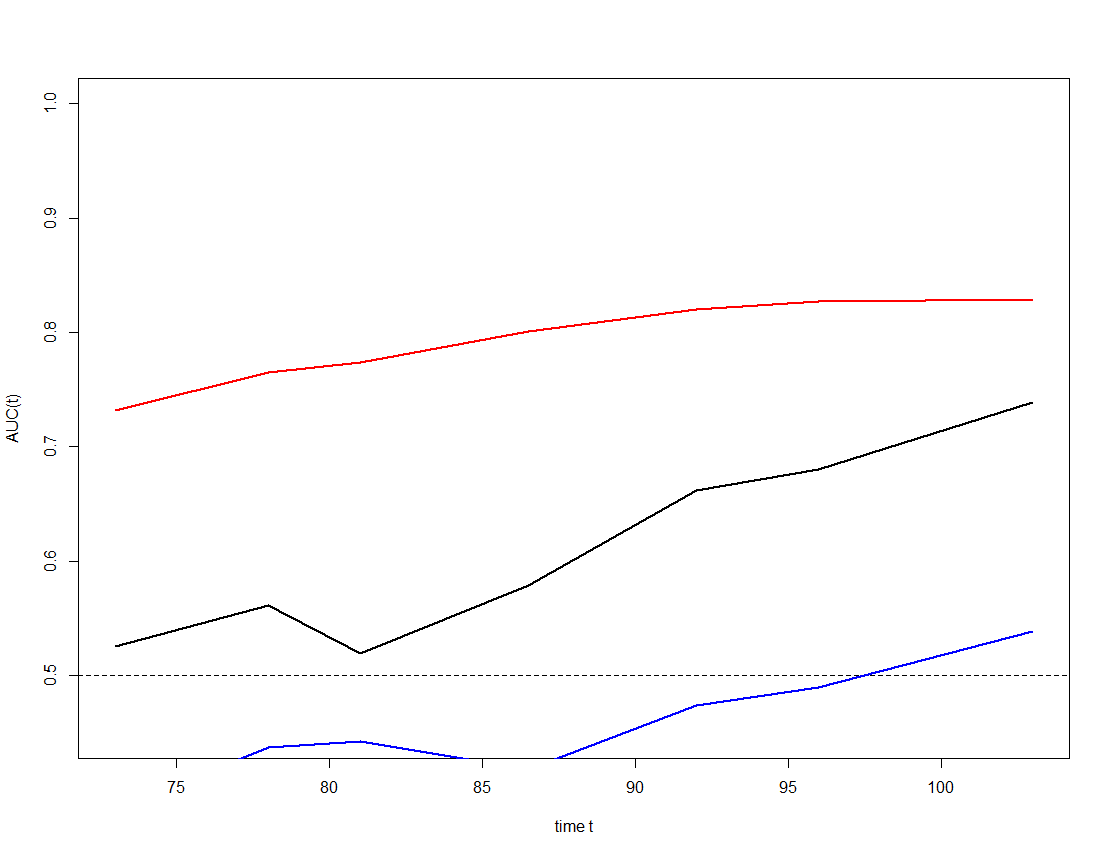


**Figure S9**

**Metastatic PFS**

**Time Dependent Receiver Operator Characteristic Curves**

**A**

**B**

**Biochemical PFS**


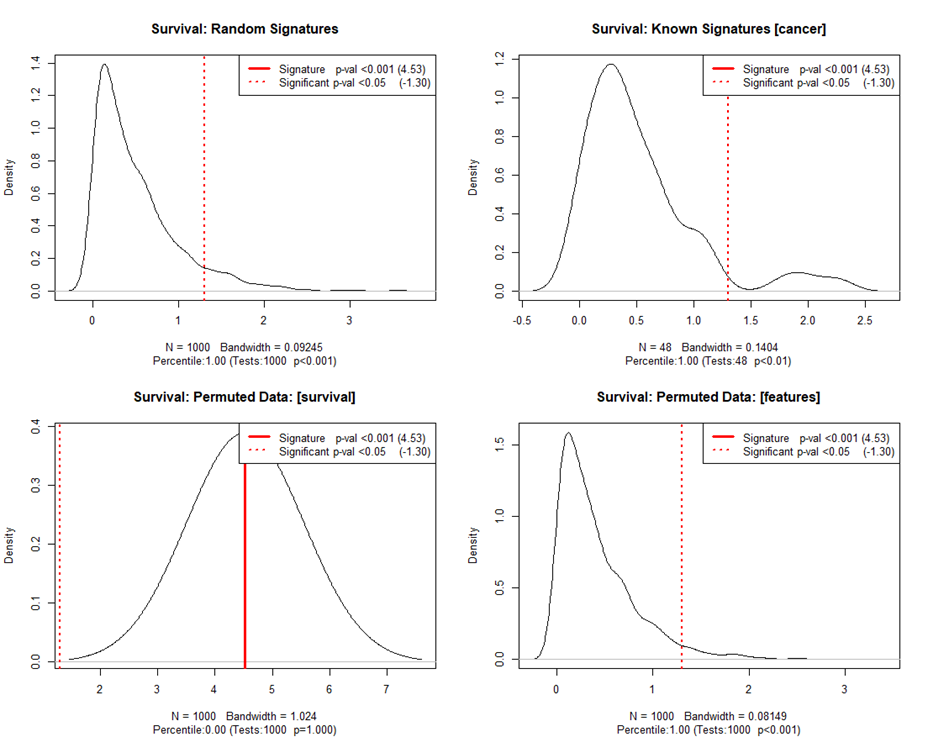


**Figure S10**


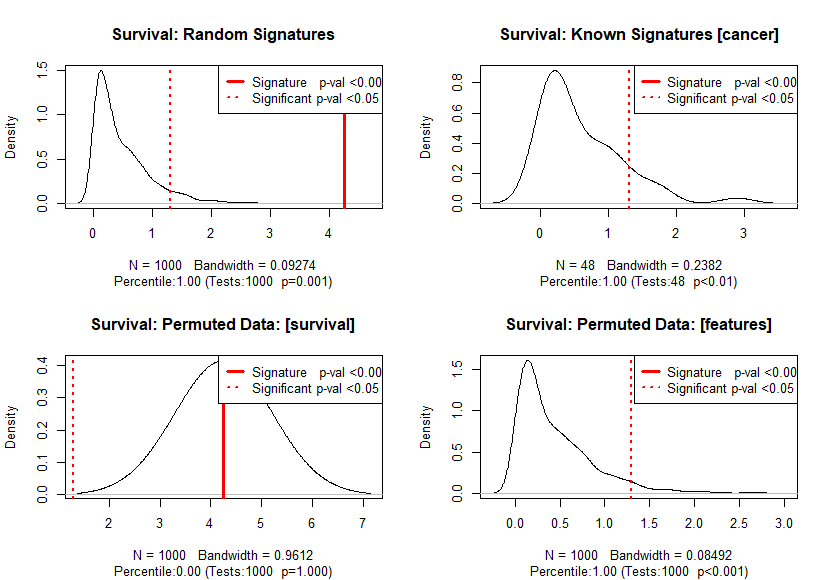


**Figure S11**


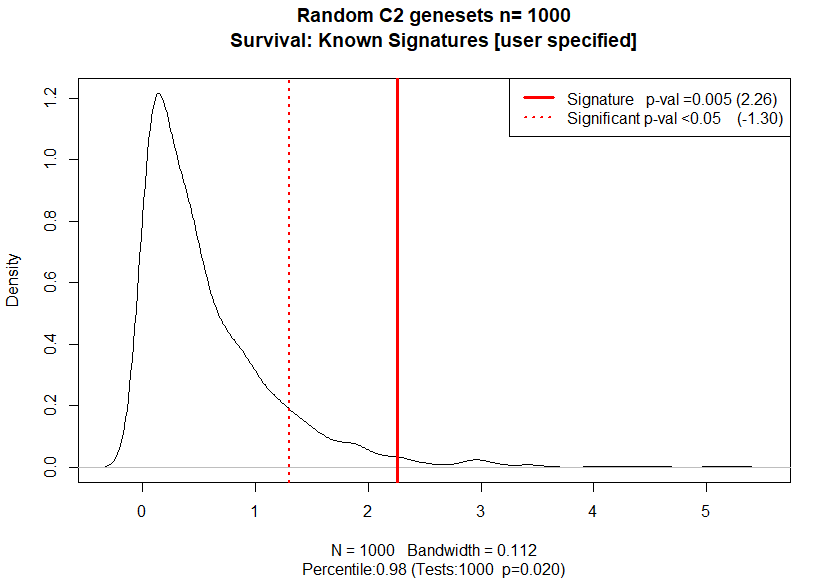

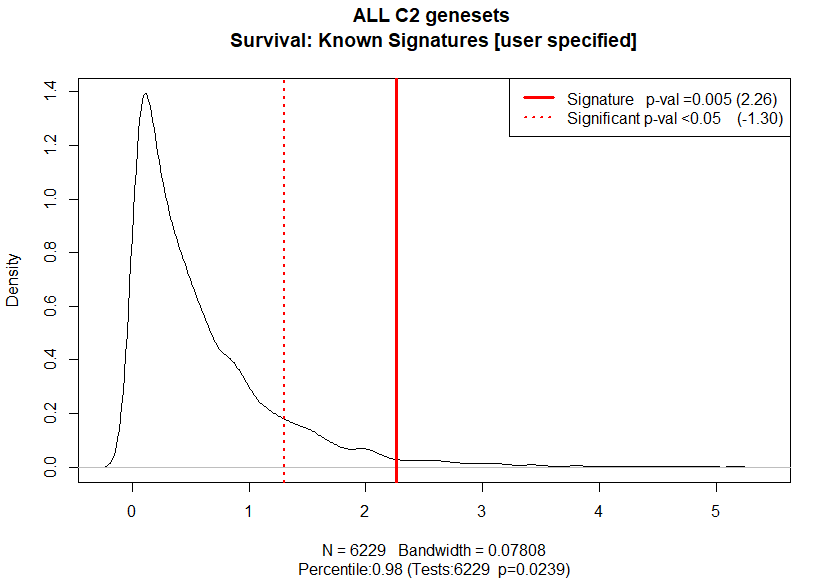


**Figure S12**


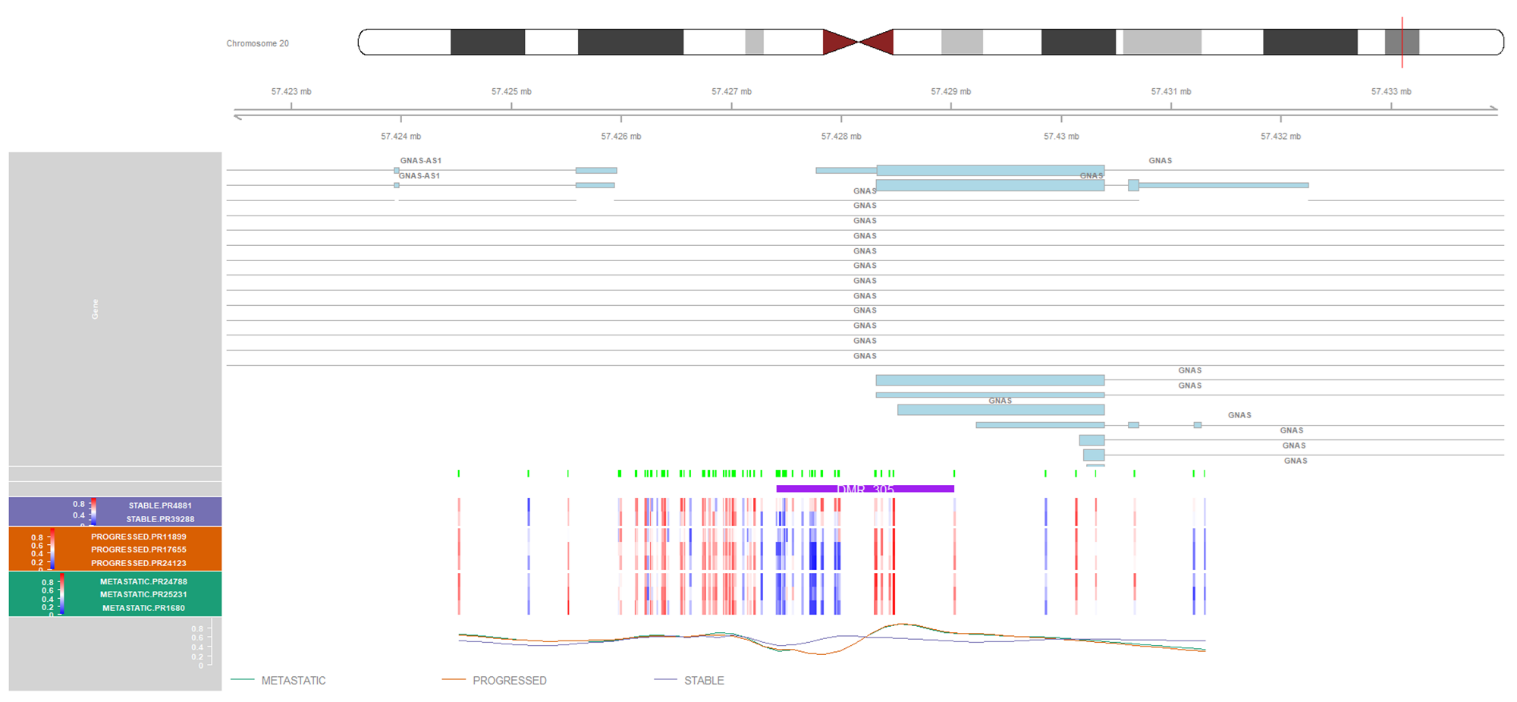


**Figure S13**


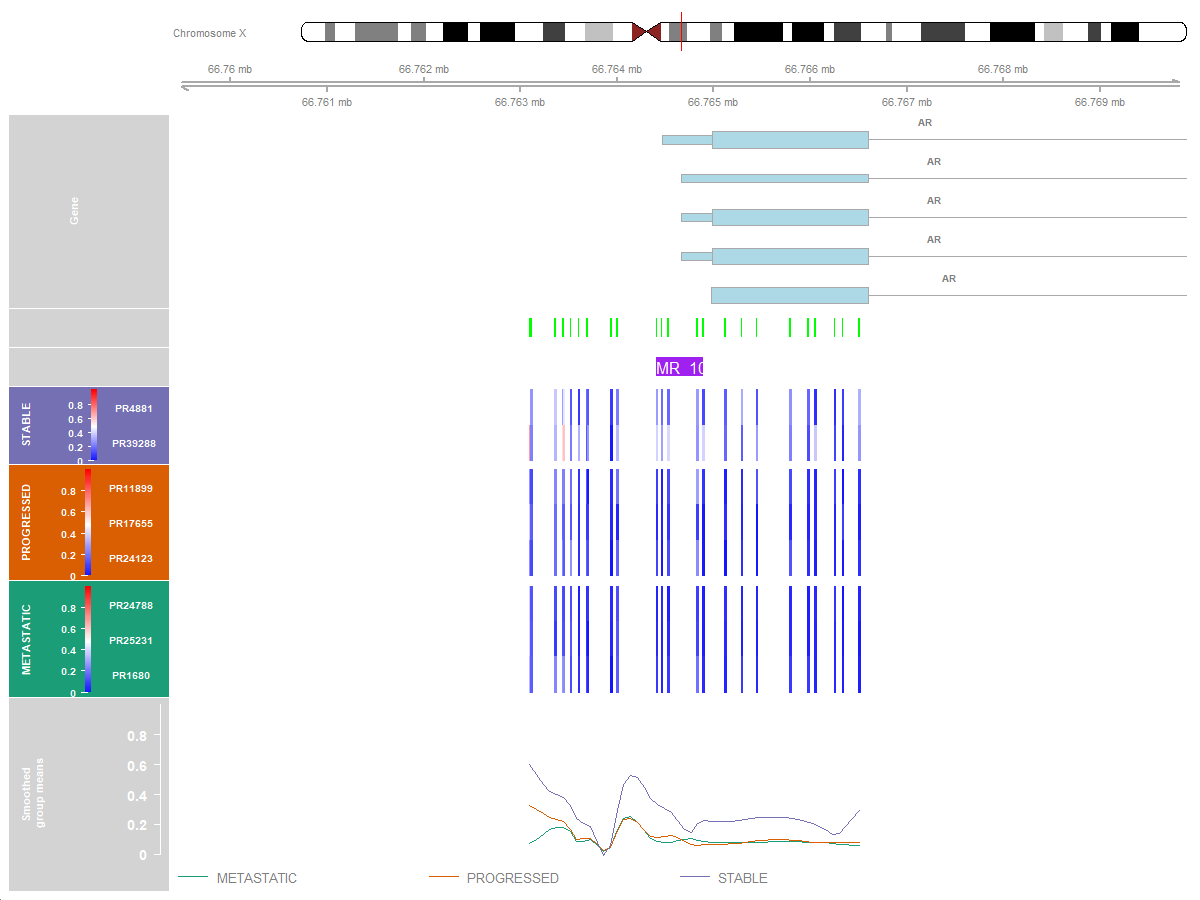

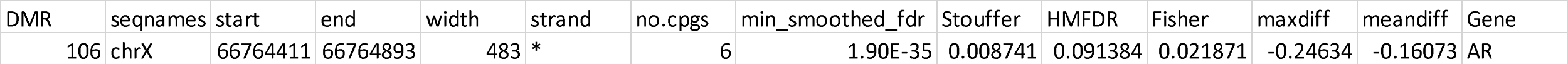


**Figure S14**


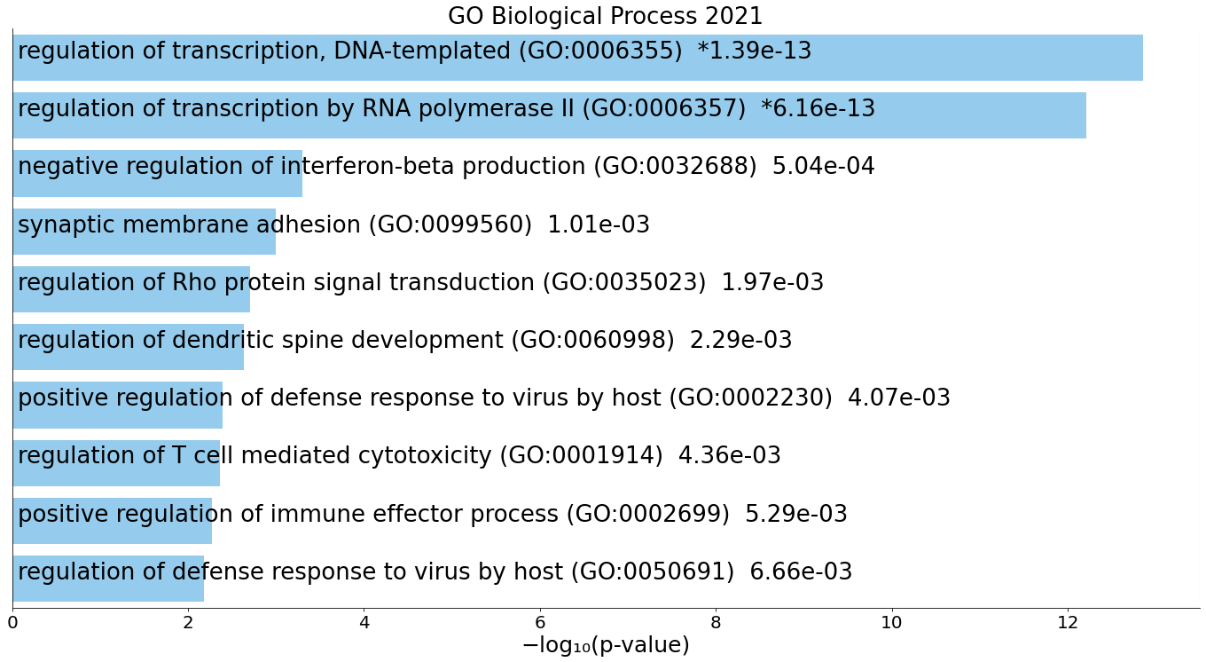


**Figure S15**


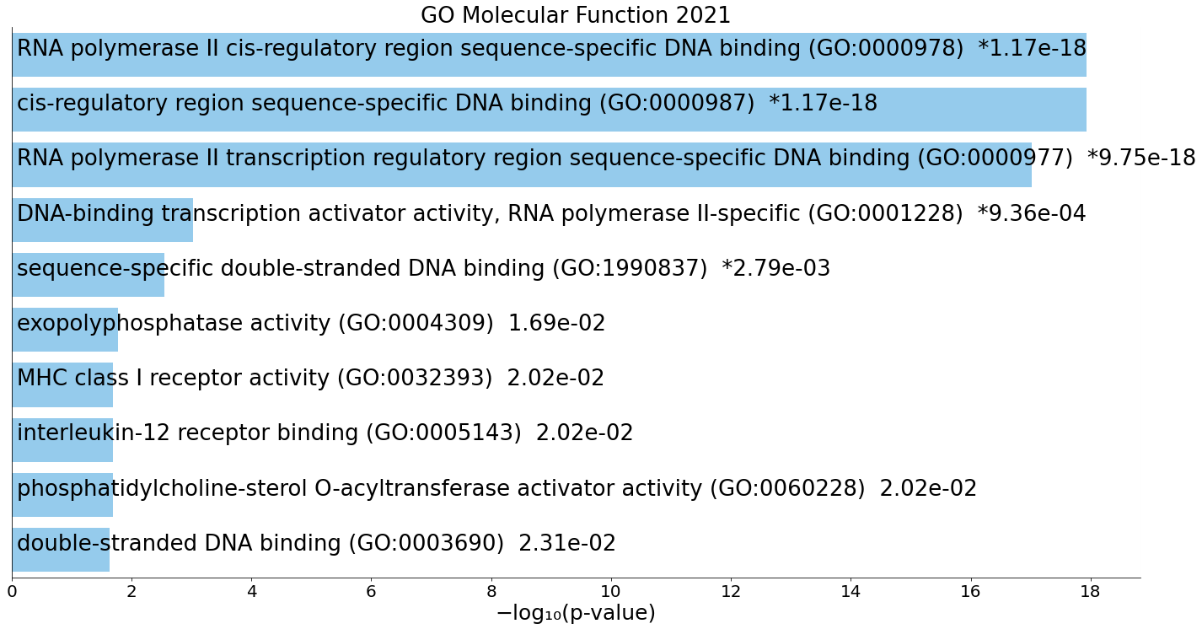


**Figure S16**


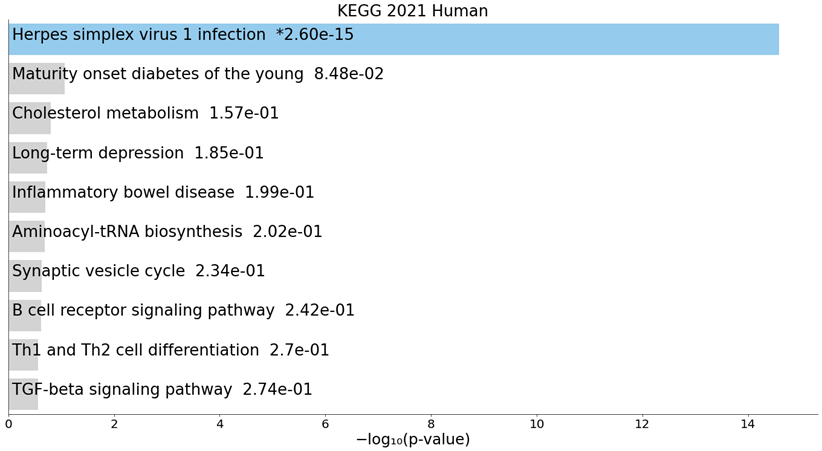


**Figure S17**


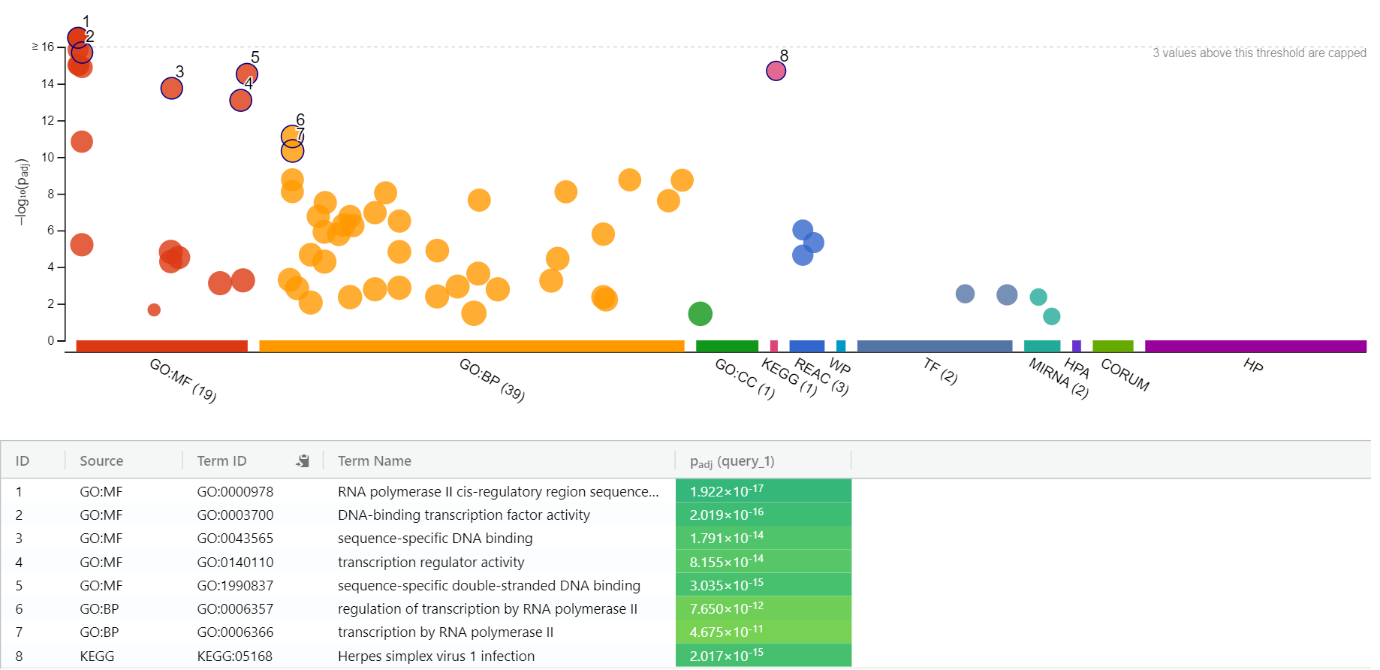


**Figure S18**


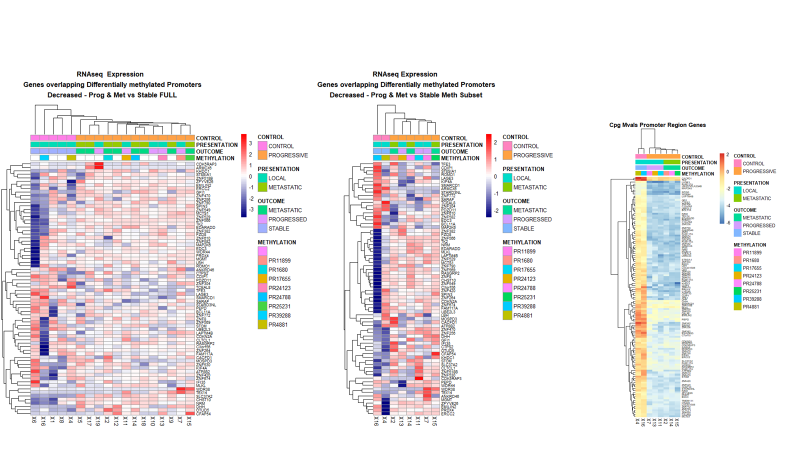


**Figure S19**

**A**

**B**

**C**


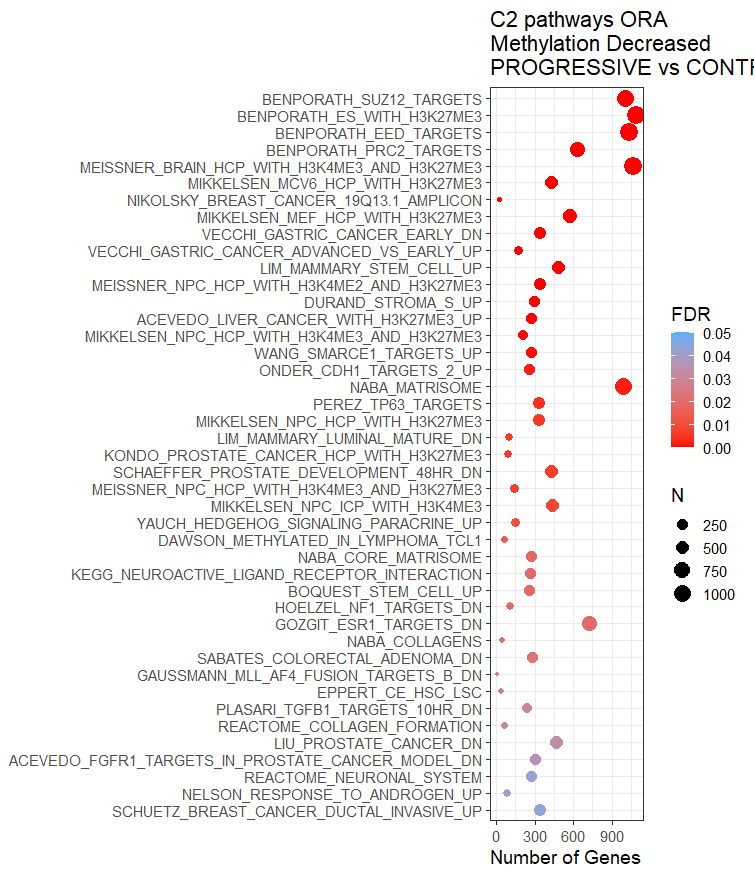

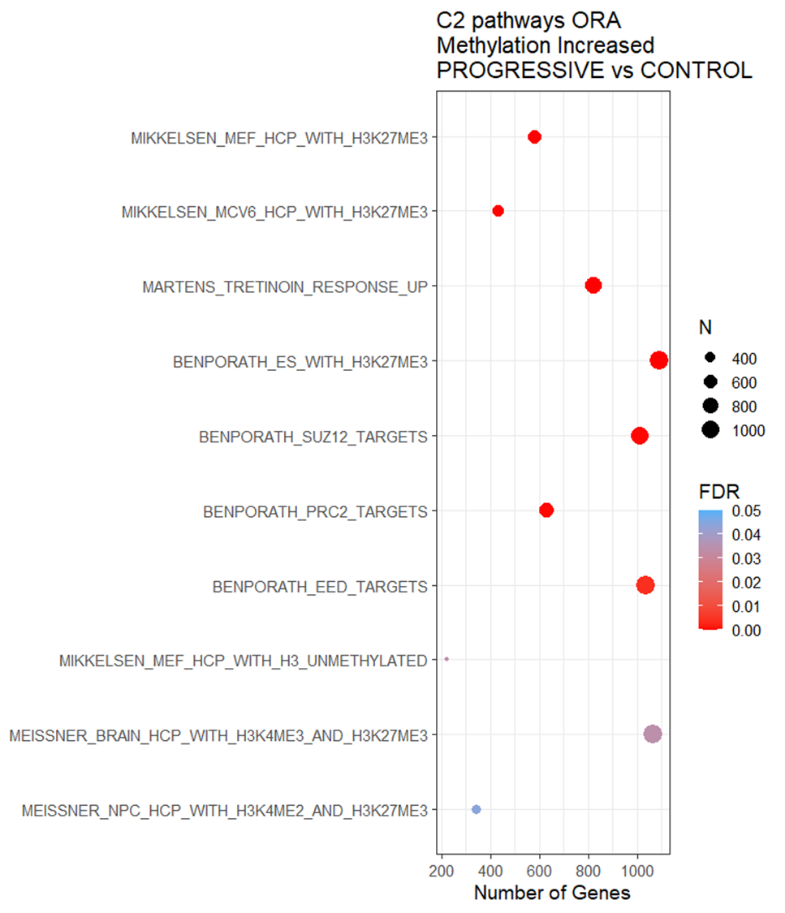


**Figure S20**

**A**

**B**


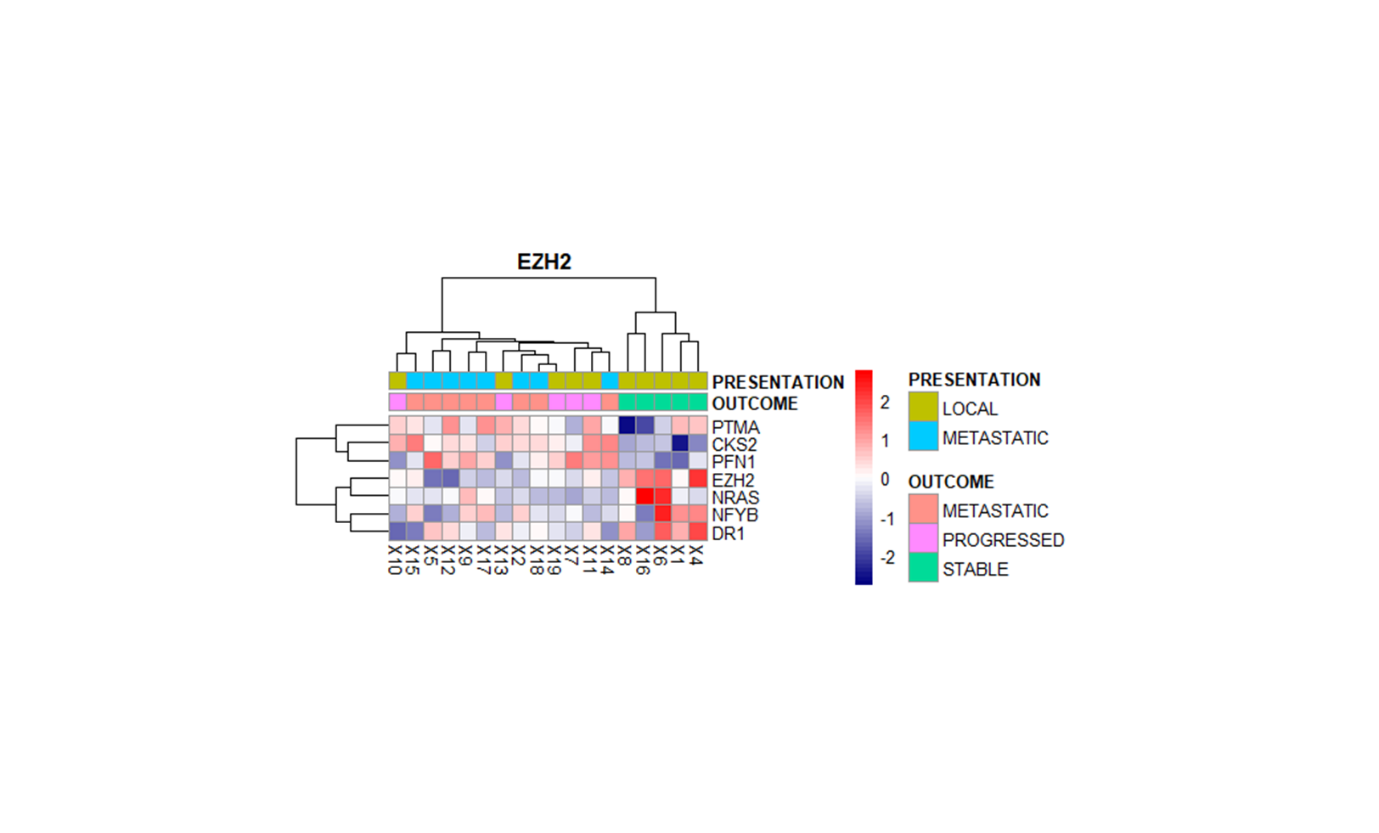


**Figure S21**


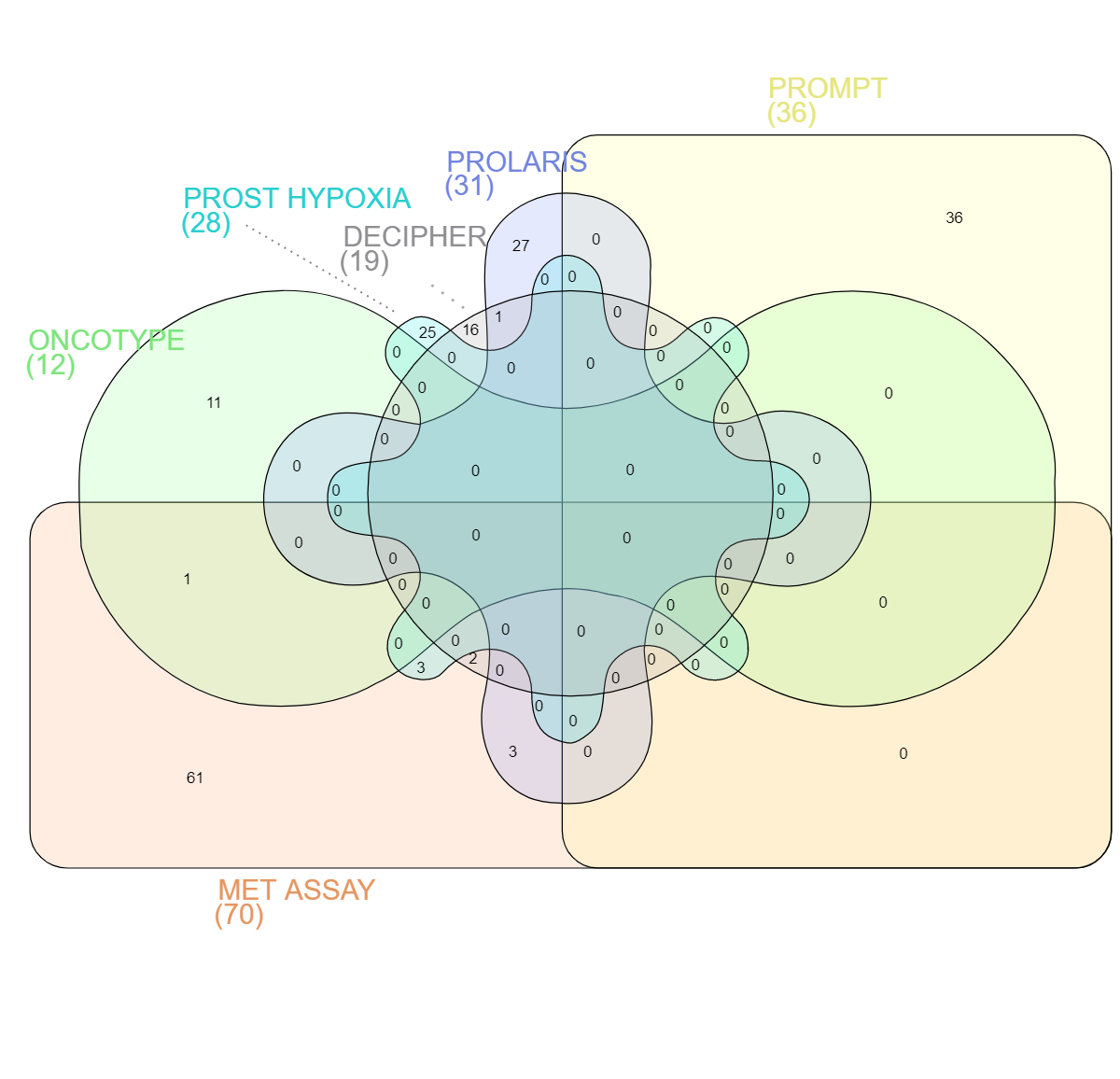


**Figure S22**
