# Supplementary figures and images for: Molecular analysis of archival diagnostic prostate cancer biopsies identifies genomic similarities in cases with progression post‐radiotherapy, and those with de novo metastatic disease
Source: Prostate. 2024 Apr 23;84(10):977–90. doi: 10.1002/pros.24715 (PMC11253896; doi:10.1002/pros.24715)

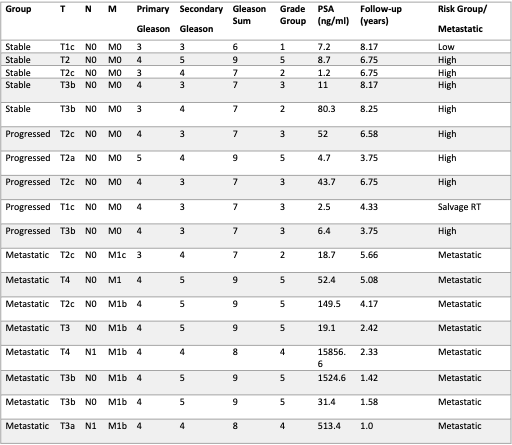


**Table S1**


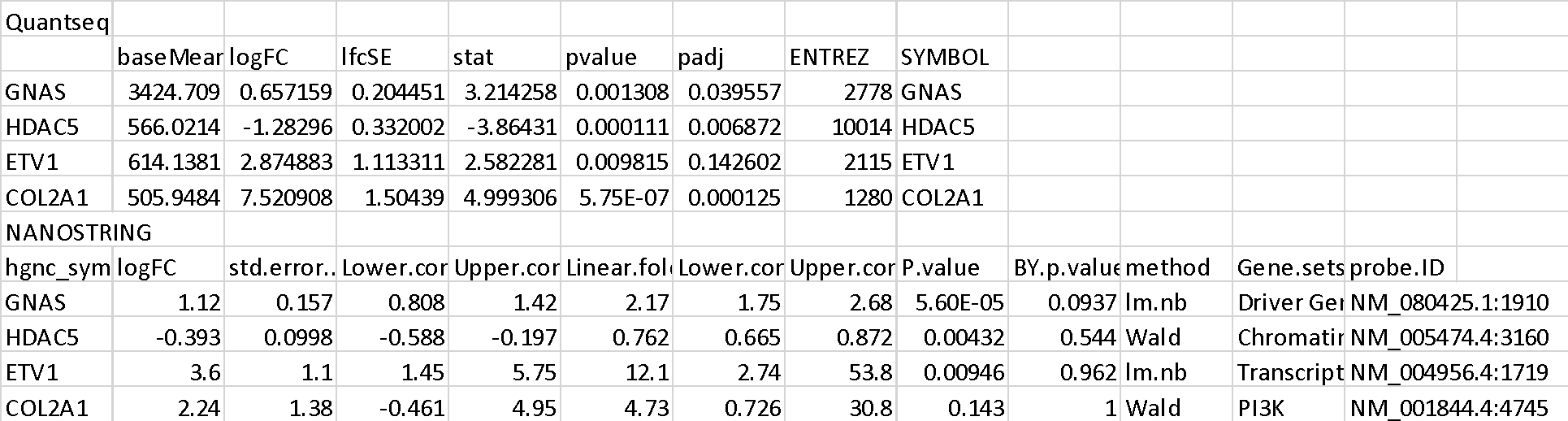


**Table S2**

**A**

**B**


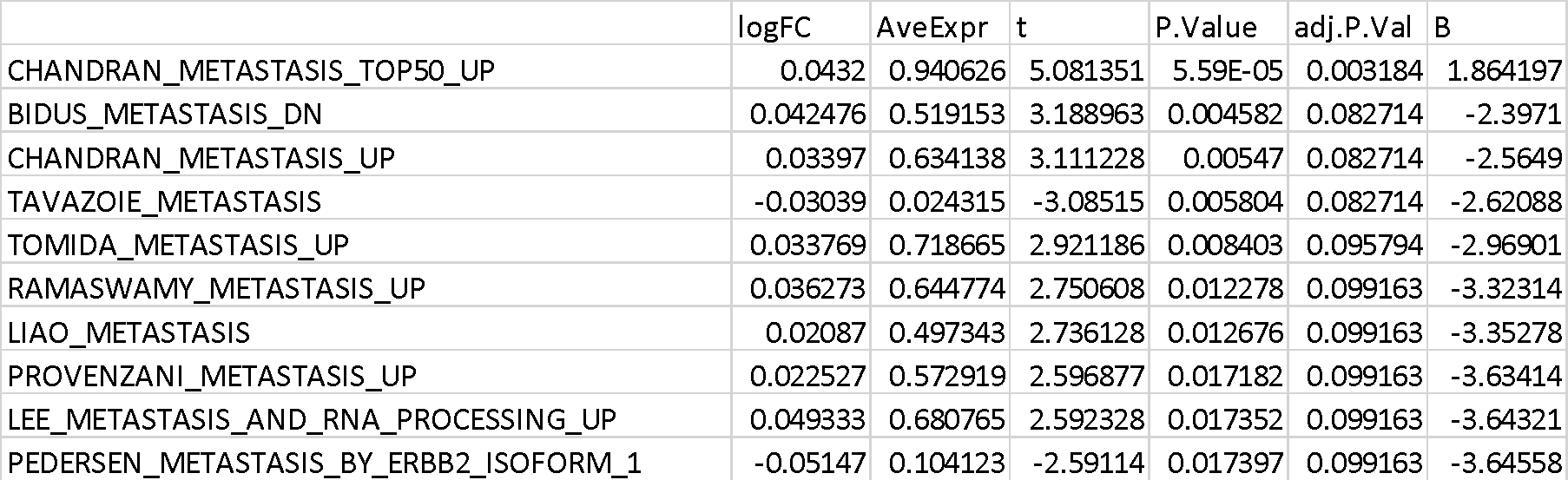


**Table S3**


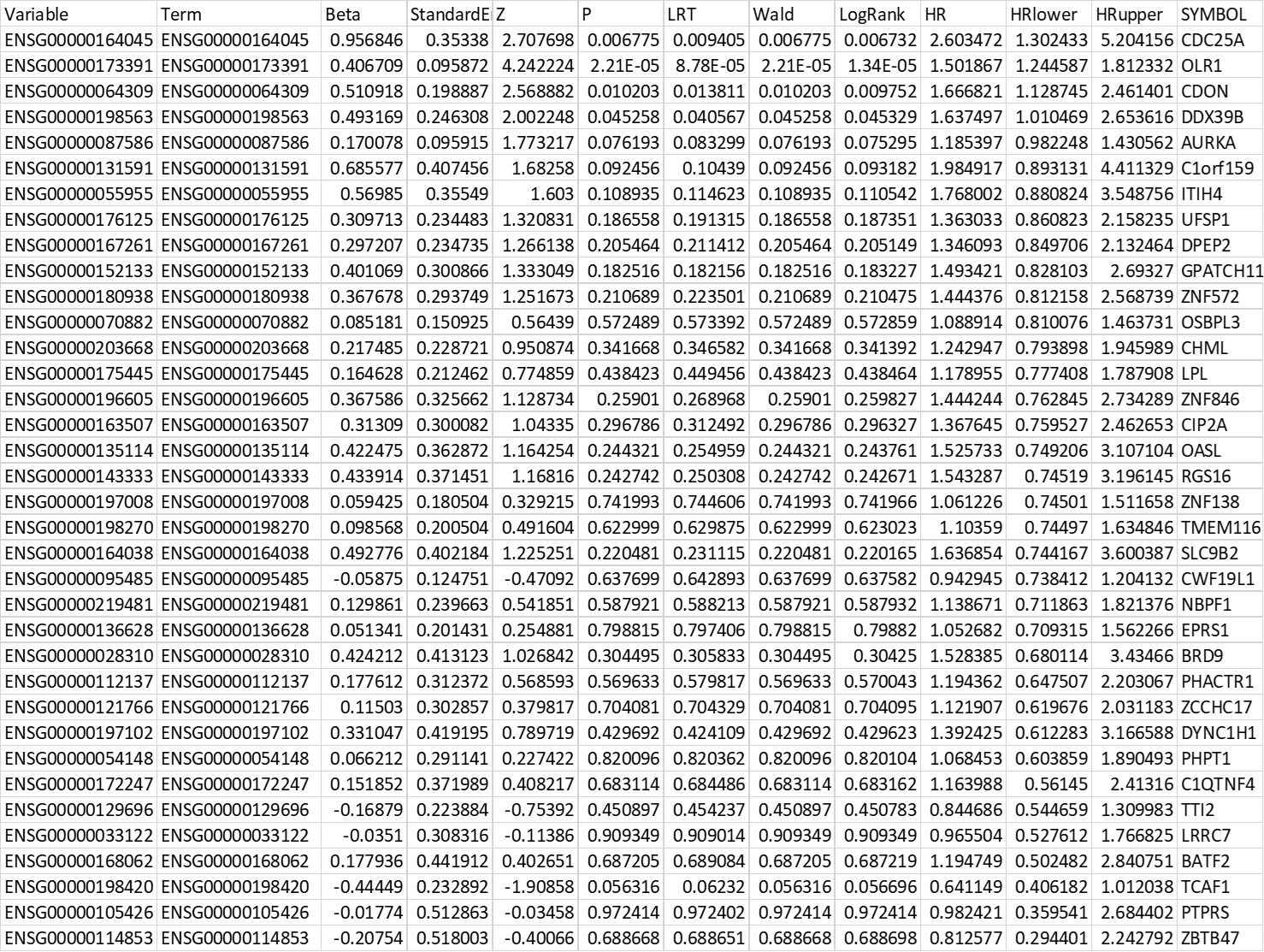


**Table S4**


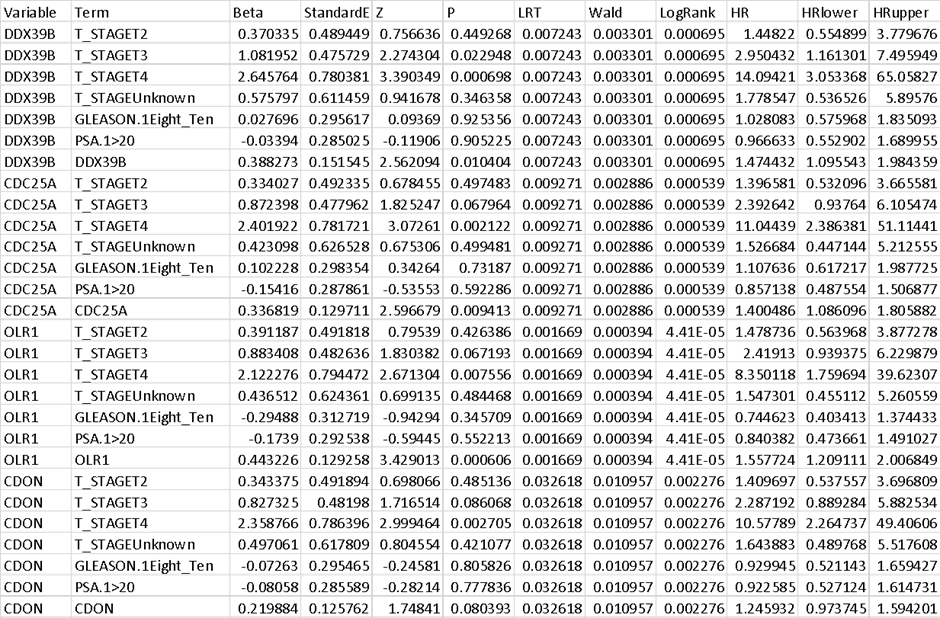


**Table S5**

Supplement: Supplementary file 2 — Supporting information. [file PROS-84-977-s002.docx]
