# Supplementary material for: Molecular analysis of archival diagnostic prostate cancer biopsies identifies genomic similarities in cases with progression post‐radiotherapy, and those with de novo metastatic disease
Source: Prostate. 2024 Apr 23;84(10):977–90. doi: 10.1002/pros.24715 (PMC11253896; doi:10.1002/pros.24715)
Supplement: Supplementary file 3 — Supporting information. [file PROS-84-977-s001.docx]

**Supplementary Methods:**

**Gene Expression & Methylation Analysis**

3’RNAseq FastQ files were concatenated for each patient, and polyA and llumina adapter trimming (AGATCGGAAGAGC) was performed using trimmomatic (version 0.25), prior to alignment with STAR (2.7.7a) to Hg38 (GRCh38.95), with featurecounts used to generate counts exact parameters are available upon request. Filtering of lowly expressed genes was performed, followed by differential expression analysis using DESEQ2 (version 1.26.0) [20]. Over-representation analysis was performed using Clusterprofiler, enrichr and gprofiler. The gene set variation analysis GSVA (version 1.34.0) package was used to perform single sample gene set enrichment analysis (ssGSEA) [21]. C2 Curated genesets (c2.all.v7.2.entrez.gmt) were used from Molecular Signatures Database (MSigDB) website (<https://www.gsea-msigdb.org/gsea/msigdb>).

For DNA methylation analysis, QC, filtering of poor performing probes, cross-reactive probes and normalization was performed prior to differential methylation analysis using minfi and MissMethyl package (version 1.32.0). The manifest file “infinium.methylationepic.v.1.0.b5” was used. DMRcate package (2.0.7) was used for differential methylation and Granges for visualization, MissMethyl (version 1.32.0) was used for pathway analyses.

**Additional Analysis**

Sequencing and clinical data from the external dataset GSE116918 were downloaded from Gene Expression Omnibus (GEO available at <https://www.ncbi.nlm.nih.gov/geo/>). Probes were filtered to only include non-overlapping exonic probes, and multiple probes were merged to the mean value per gene.

To overcome technology platform differences, as GSE116918 utilized a different sequencing technology (microarray) to those used in this study 3’RNAseq, mean values of fully exonic probes for the relevant genes were used from the GSE116918 dataset.

NanoString probes are designed to span exons, such that only mature mRNAs are counted. The Quantseq method sequences mature transcripts with polyA tails, and this was used with the FeatureCounts and Subread package for quantification and identifications of alternate transcripts. Survminer and Survival packages were used for survival analysis with cox proportional hazards models utilizing known clinicopathological features, staging and Gleason score. Survival analysis was performed using a Cox proportional hazards model comprising clinicopathological features and expression of selected genes, followed by estimation of time-dependent Receiver Operator Characteristics (ROC) using TimeROC (package 0.4). KMunicate (0.2.0) and survival (3.2-3) packages were used for survival analysis, with extended risk tables for time to event analysis.

R sessionInfo() is provided in the Package Summary file.

**Figure S1**. Signature Summary scores produced by SigQC for previously validated signatures in prostate cancer (Prostate Hypoxia, Prolaris, Oncotype and Decipher) in the PROMPT dataset compared to TCGA Prostate PanCancer and Jain Radiotherapy datasets. Overall signature performance is comparable in the PROMPT dataset compared to previously published datasets.

**Figure S2**. Intra-signature correlation generated by SigQC for previously validated signatures in prostate cancer (Prostate Hypoxia, Prolaris, Oncotype and Decipher) in the PROMPT dataset compared to TCGA Prostate PanCancer and Jain radiotherapy datasets. Intra-Signature correlation is comparable in the PROMPT dataset compared to previously published datasets.

**Figure S3**. Signature expression heatmaps. Expression of previously validated prostate cancer signatures in PROMPT dataset; Prostate Hypoxia (**A**), Decipher (**B**), Prolaris (**C**), Oncotype (**D**).

**Figure S4**. Over-representation analysis of genes increased in progressed versus stable prostate cancer patients post-RRT (padj <0.01) (**A**). A network plot visualizing the enriched genes (**B**).

**Figure S5**. Over-representation analysis of genes increased in metastatic versus stable prostate cancer patients post-RRT (padj <0.05) (**A**). A network plot visualizing the enriched genes (**B**).

**Figure S6**. Single sample GeneSet Enrichment Analysis (ssGSEA) pathways analysis of progressed & metastatic cases versus stable cases post-RRT. ssGSEA identifies multiple pathways from MSigDB C2: curated gene sets, significantly different between progressed and metastatic cases versus stable cases post-RRT. 3 Pathways are enriched in stable cases, 11 pathways are enriched in progressed and metastatic cases.

**Figure S7**. Overlap of genes in significant single sample GeneSet Enrichment Analysis (ssGSEA) pathways, comparing progressed and metastatic cases versus stable cases. Overlap is measured between 0 (0%) and 1 (100%).

**Figure S8**. Univariable analysis identifies 4 genes having a lower HR value >1 in the Jain Radiotherapy Cohort.4-gene (mean per gene - median cut-off for cohort) **(A)**. A Cox proportional hazards model analysis of biochemical progression-free survival was performed **(B)**. Forest plot of the cox proportional hazard model for biochemical progression-free survival, incorporating clinicopathological features (T-stage, Gleason score and PSA using clinically relevant cut-points) and high/low expression cohorts (mean per gene - median value threshold for dividing cohort) of the 4 gene signature in the Jain radiotherapy cohort **(B)**.

**Figure S9.** Time dependent Receiver Operator Characteristic curves for biochemical **(A)** and metastatic **(B)** progression-free survival in the Jain radiotherapy dataset, for the 4 gene signature, PSA and Gleason sum score.

**Figure S10**. SigCheck comparison of random signatures, cancer signatures, and survival and feature permutations for biochemical progression-free survival in the Jain dataset. The vertical red dotted line shows where a "significant" result (p=0.05) would lie relative to the background distribution.

**Figure S11**. SigCheck comparison of random signatures, cancer signatures, and survival and feature permutations for metastatic progression-free survival in the Jain dataset. The vertical red dotted line shows where a "significant" result (p=0.05) would lie relative to the background distribution.

**Figure S12**. SigCheck comparison of subset and all curated cancer signatures (MsigDB) compared to PROMPT signature for metastatic progression-free survival in the Jain dataset. The vertical red dotted line shows where a "significant" result (p=0.05) would lie relative to the background distribution.

**Figure S13**. Differentially Methylated Region plot for the *GNAS* gene, visualizing differential methylation between progressed and metastatic cases versus stable cases post-RRT. Genomic co-ordinates and proximal coding regions (top), and heatmap and mean methylation plots (bottom), are illustrated.

**Figure S14**. Differentially Methylated Region plot for the *Androgen Receptor* gene, visualizing differential methylation between progressed and metastatic cases versus stable cases post-RRT. Genomic co-ordinates and proximal coding regions (top), and heatmap and mean methylation plots (bottom), are illustrated.

**Figure S15**. Bar-plot of over-representation analysis of Chromosome 19 (enrichr) GO Biological Process pathways, and Differentially Methylated Genes, between progressed and metastatic cases versus stable cases.

**Figure S16**. Bar-plot of over-representation analysis of Chromosome 19 (enrichr) GO Molecular Function pathways, and Differentially Methylated Genes, between progressed and metastatic cases versus stable cases.

**Figure S17**. Bar-plot of over-representation analysis of Chromosome 19 (enrichr) KEGG pathways, and Differentially Methylated Genes, between progressed and metastatic cases versus stable cases.

**Figure S18**. Over-representation analysis of all pathways for Chromosome 19 (gprofiler).

**Figure S19**. Heatmap analysis of RNA expression of differentially hypo-methylated genes at promoter in the full cohort, methylation subset, and cpg Mvals for methylation. Quantseq RNA expression analysis heatmap of genes with differentially hypo-methylated promoter regions between progressed and metastatic cases versus stable cases in the whole cohort (**A**). Quantseq RNA expression analysis heatmap of genes with differentially hypo-methylated promoter regions between progressed and metastatic cases versus stable cases in the methylation analysis cohort (**B**). Methylation (Mvals) heatmap of Cpgs in promoter regions differentially methylated between progressed and metastatic cases versus stable cases (**C**).

**Figure S20**. Dot-plot over-representation analysis of hypomethylated (**A**) and hypermethylated (**B**) C2 Curated Pathway (MSigDB) genes in progressed and metastatic cases versus stable cases post-RT.

**Figure S21**. Heatmap of EZH2 expression, and expression of EZH2 target genes, from the Quantseq data, demonstrating significant differential expression between progressed and metastatic cases versus stable cases (padj<0.05).

**Figure S22**. Signature Genelist overlap.

**Table S1**. Baseline clinicopathological characteristics of the cohort.

**Table S2**. Overlap of significantly differentially expressed genes between progressed and stable samples in both Quantseq (padj <0.2) and Nanostring (p<0.05) analysis. Genes increased in progressed versus stable cases in Quantseq and Nanostring include GNAS, ETV1, COL2A1. HDAC5 expression is decreased in progressed versus stable cases in Quantseq and Nanostring. LogFC - Log Fold Change; lfcSE - standard error; padj - adjusted p-value (**A**). A per gene univariable cox proportional hazards analysis was performed for these genes using the Jain Radiotherapy dataset (**B**).

**Table S3**. Guided single sample GeneSet Enrichment Analysis (ssGSEA) of MSigDB C2: curated metastasis gene set signatures in the Jain dataset. LogFC - Log Fold Change; AveExpr - Average Expression; t = t statistic; P.Value - p-value; adj.P.Val - adjusted p-value; B - B statistic.

**Table S4**. Cox proportional hazard analysis of genes independently associated with biochemical progression-free survival.

**Table S5**. 4 gene (mean per gene - median cut-off for cohort) cox proportional hazards and clinical variables.
